# Breast cancer and background parenchymal enhancement at breast magnetic resonance imaging: a meta-analysis

**DOI:** 10.1186/s12880-021-00566-8

**Published:** 2021-02-19

**Authors:** Na Hu, Jinghao Zhao, Yong Li, Quanshui Fu, Linwei Zhao, Hong Chen, Wei Qin, Guoqing Yang

**Affiliations:** Department of Radiology, Suining Central Hospital, Suining, 629000 Sichuan China

**Keywords:** Dynamic contrast-enhanced magnetic resonance imaging, Breast magnetic resonance imaging, Breast cancer, Background parenchymal enhancement

## Abstract

**Background:**

The background parenchymal enhancement at breast magnetic resonance imaging use to predict breast cancer attracts many searchers to draw a possible relationship. However, the results of their relationships were conflicting. This meta-analysis was performed to assess breast cancer frequency associations with background parenchymal enhancement.

**Methods:**

A systematic literature search up to January 2020 was performed to detect studies recording associations between breast cancer frequency and background parenchymal enhancement. We found thirteen studies including 13,788 women at the start with 4046 breast cancer. We calculated the odds ratio (OR) and the 95% confidence intervals (CIs) between breast cancer frequency and background parenchymal enhancement by the dichotomous technique with a random or fixed-effect model.

**Results:**

Women with minimal or mild background parenchymal enhancement at breast magnetic resonance imaging did not have any risk of breast cancer compared to control women (OR, 1.20; 95% CI 0.54–2.67). However, high background parenchymal enhancement at breast magnetic resonance imaging (OR, 2.66; 95% CI 1.36–5.19) and moderate (OR, 2.51; 95% CI 1.49–4.21) was associated with a significantly higher rate of breast cancer frequency compared to control women.

**Conclusions:**

Our meta-analysis showed that the women with high and moderate background parenchymal enhancement at breast magnetic resonance imaging have higher risks, up to 2.66 fold, of breast cancer. We suggest that women with high or moderate background parenchymal enhancement at breast magnetic resonance imaging to be scheduled for more frequent follow-up and screening for breast cancer to avoid any complications.

## Background

The level of normal fibroglandular tissue that enhances the breast magnetic resonance imaging is recognized as background parenchymal enhancement [1, 2]. Background parenchymal enhancement is related to females hormones and it is reduced post-menopause due to the reduction in these hormones [3].

The American college of radiology breast imaging reporting, and data system, or breast imaging reporting, and data system lexicon categorized background parenchymal enhancement [2]. Their categories are based on the quantity of fibroglandular tissue, and not the entire breast volume. They are minimal, mild, moderate, and high background parenchymal enhancement [4].

The background parenchymal enhancement data is highly available due to the high number of prescribed breast magnetic resonance imaging for screening and diagnosing breast cancer. Breast magnetic resonance imaging is better to be scheduled at days 7–14 through the follicular phase of women's menstrual cycles since background parenchymal enhancement is more noticed in the luteal phase of the menstrual cycle [5, 6].

Recently, high background parenchymal enhancement was considered as a marker of higher risk of breast cancer in women at high risk e.g. women using an adjuvant endocrine treatment [7–10]. Also, it was found to detect the efficacy of treatment in some other women e.g. women using neoadjuvant chemotherapy [11].

So, background parenchymal enhancement can be used to improve the early discovery and inhibition of breast cancer [12]. Though, its associations with breast cancer data are still conflicting. Our meta-analysis aimed to assess the breast cancer frequency association with background parenchymal enhancement.

## Methods

The present study complied with the meta-analysis of studies in the epidemiology statement [13], which was performed following an organized protocol.

### Study detection

**Fig. 1:**
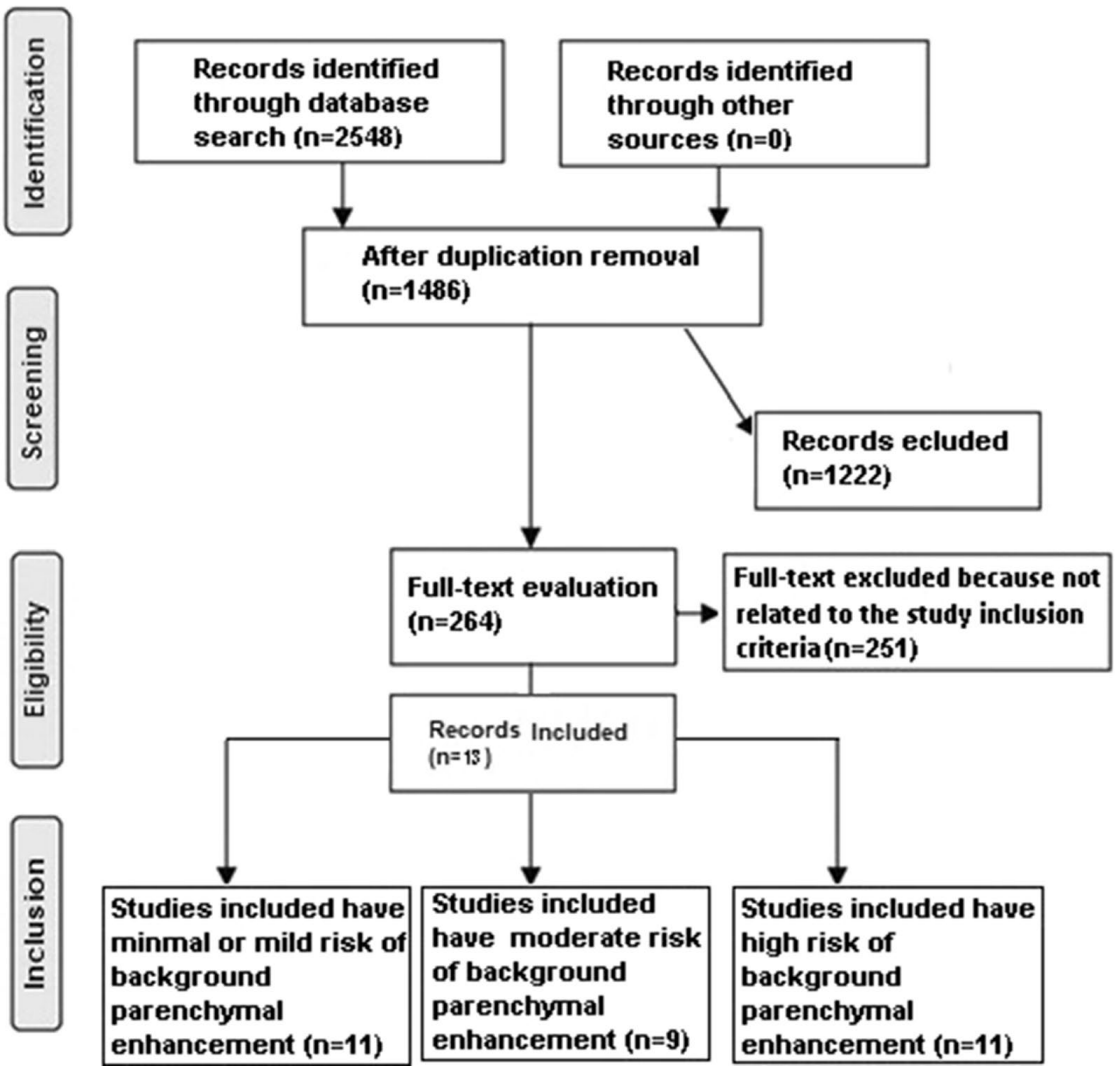
Schematic diagram of the study process

Included studies were human studies in English language with the association between breast cancer frequency and background parenchymal enhancement in women with and without breast cancer. Excluded studies were commentary, review articles and studies with no measure of an association. Figure 1 shows a schematic diagram of the study process. The articles were integrated into the meta-analysis when the following inclusion criteria were met:

1. The study was a randomized controlled trial or retrospective study.
2. The target population is women with and without breast cancer.
3. The intervention program was based on assessments of background parenchymal enhancement.
4. The study included women with and without breast cancer.

### Identification

A protocol of search strategies was prepared according to the PICOS principle [13], and we defined it as follow: P (population): women with and without breast cancer; I (intervention/exposure): assessments of background parenchymal enhancement; C (comparison): women with compared to without breast cancer; O (outcome): recurrence of giant cell tumor of bone; and S (study design): no restriction [14].

A systematic search was performed in OVID, Embase, Cochrane Library, PubMed, and Google scholar till January 2020, using a blend of keywords “dynamic contrast-enhanced magnetic resonance imaging, magnetic resonance imaging, background parenchymal enhancement and breast cancer”. We gathered all studies detected in an EndNote file, we removed any duplication found and revised the title and abstracts to eliminate studies that did not show any association between breast cancer frequency and background parenchymal enhancement in breast magnetic resonance imaging, based on our inclusion and exclusion criteria. The remaining studies were examined for any possible association.

### Screening

Data were abridged into a stranded form [14]. If one study contained different data depending on the assessment of the breast cancer frequency and background parenchymal enhancement, they were extracted separately. The selected studies' risk of bias was assessed using the quality in prognosis studies tool, which assesses bias and validity using 6 domains: participation, attrition, prognostic factor measurement, confounding measurement, and account, outcome measurement, and analysis and reporting [15].

### Eligibility and inclusion

The main result concentrated on the breast cancer frequency association with background parenchymal enhancement. Background parenchymal enhancement outcomes were compared in women with and without breast cancer and the overall summary result were prepared to perform the statistical analysis.

### Statistical analysis

We used the dichotomous technique with a random-effect model or fixed-effect to compute the odds ratio (OR) and 95% confidence interval (CI). The I^2^ index was calculated; the I^2^ index is from 0 to 100%. When I^2^ was > 50%, we used the random-effect model and when it was < 50%, we used the fixed-effect model. I^2^ index of around 0%, 25%, 50%, and 75% respectively specify no, low, moderate, and high heterogeneity [16]. A subgroup analysis was made by stratifying the assessment per result as outlined previously. In this analysis, a p-value for differences between subgroups of < 0.05 was considered statistically significant. Publication bias was assessed quantitatively with the Egger regression test (publication bias considered present if *p* ≥ 0.05), and qualitatively, by visual examination of funnel plots of the logarithm of odds ratios versus their standard errors [14]. All p-values were 2 tailed. We use reviewer manager version 5.3 (The Nordic Cochrane Centre, The Cochrane Collaboration, Copenhagen, Denmark) for all calculations and graphs in this meta-analysis study.

## Results

A total of 2546 unique studies were detected, of which 13 studies fulfilled the inclusion criteria and were used in this meta-analysis study [5, 8, 9, 17–26].

The 13 studies included 13,788 women at the baseline of the study; 4046 of them were breast cancer. All studies had different background parenchymal enhancement categories in women with breast cancer.

Study size ranged from 32 to 4247 women at the baseline of the study. The number of breast cancer associated with background parenchymal enhancement ranged from 14 to 540. 9 studies reported data stratified women by minimal or mild background parenchymal enhancement (Minimal and mild were represented separately in some studies and together in some studies; consequently we decided to show them here together to avoid any inconsistency); 11 studies were with moderate background parenchymal enhancement; and 11 studies were with high background parenchymal enhancement in association with breast cancers frequency.

8 studies observed higher breast cancer frequency associated with high background parenchymal enhancement and 8 studies with moderate background parenchymal enhancement. The magnitude of the increase of breast cancer frequency was significantly higher in high background parenchymal enhancement than that in moderate background parenchymal enhancement. The effect of background parenchymal enhancement on breast cancer frequency was detected in all populations studied.

**Fig. 2:**
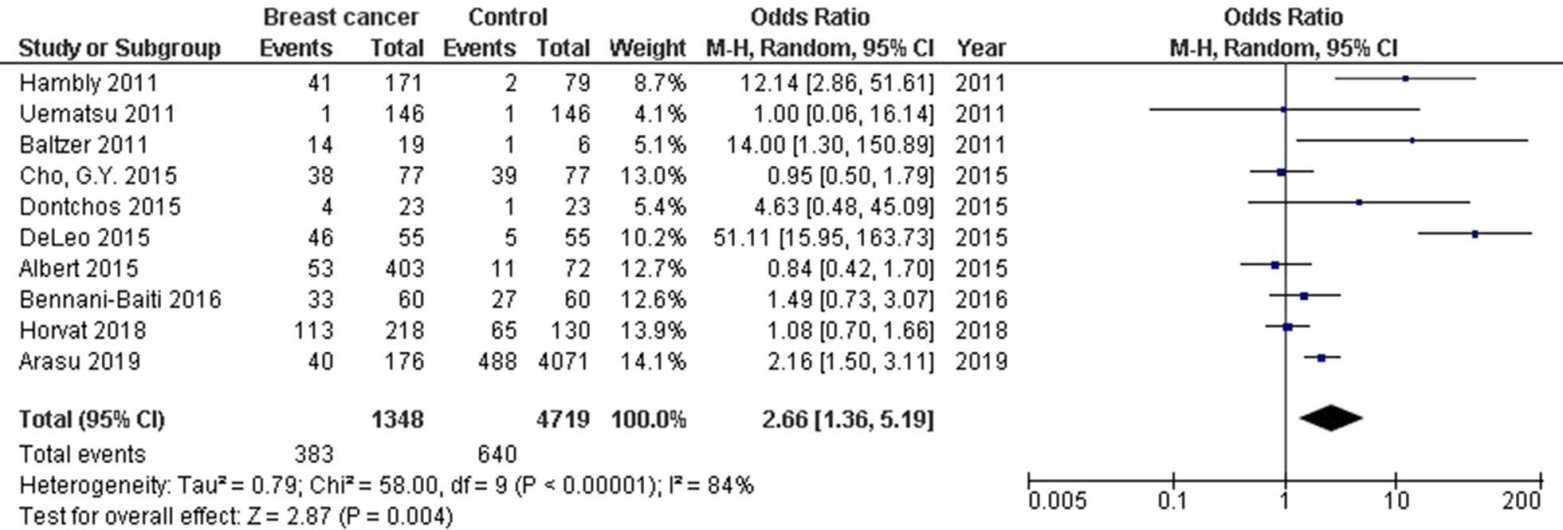
Forest plot of the high background parenchymal enhancement associated with breast cancer associated with breast cancer

**Fig. 3:**
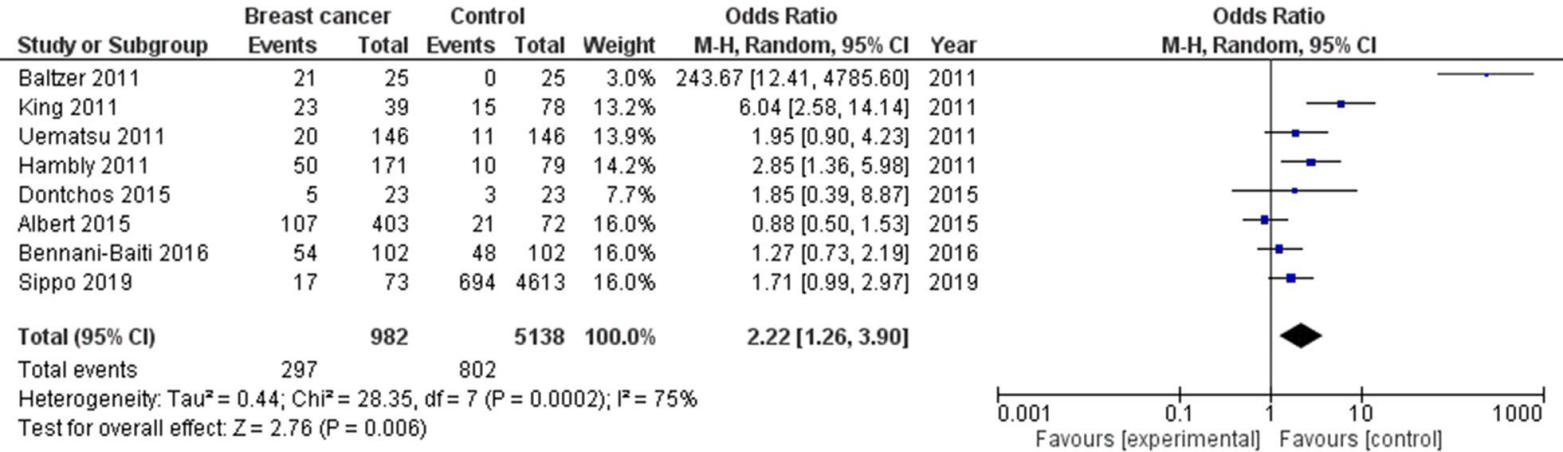
Forest plot of the moderate background parenchymal enhancement associated with breast cancer associated with breast cancer

**Fig. 4:**
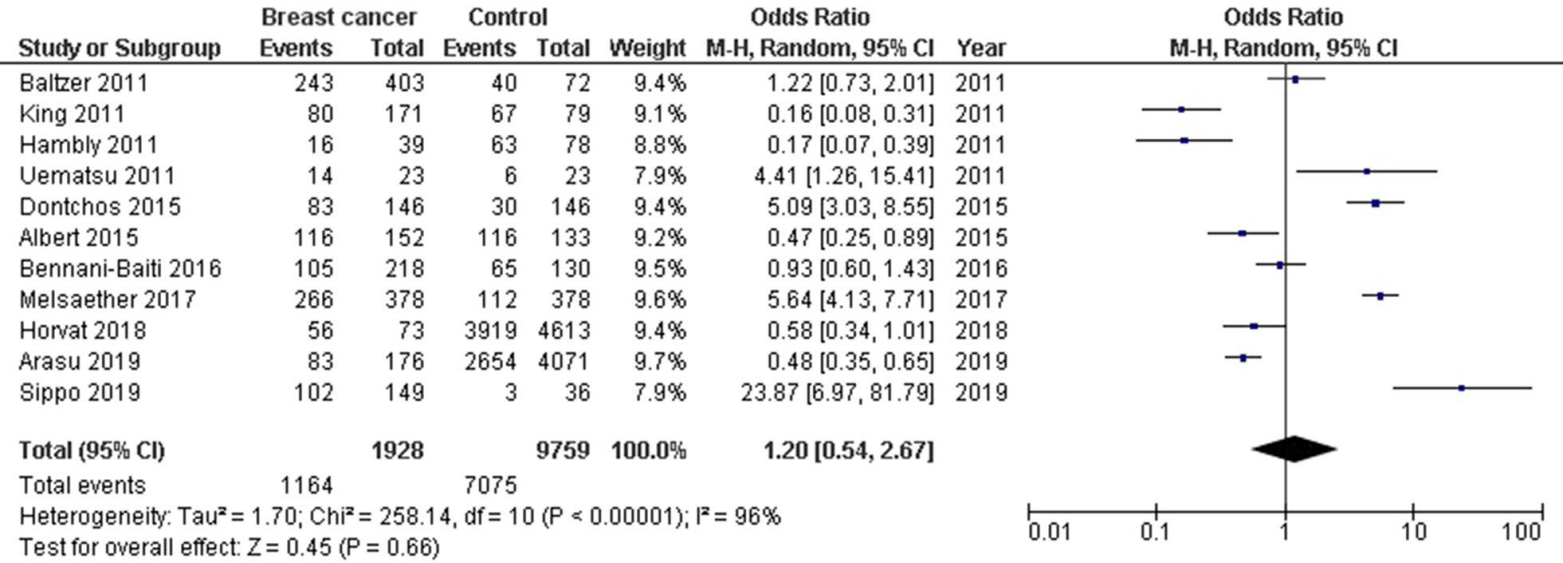
Forest plot of the minimal or mild background parenchymal enhancement associated with breast cancer

Women with high background parenchymal enhancement had significantly higher breast cancer frequency (OR, 2.66; 95% CI 1.36–5.19, *p* = 004) than that in the normal women with high heterogeneity (I^2^ = 84%) as shown in Fig. 2. Women with moderate background parenchymal enhancement category had significantly high breast cancer frequency (OR, 2.51; 95% CI 1.49–4.21, *p* < 0.001) than that in the normal women with high heterogeneity (I^2^ = 78%) as shown in Fig. 3. However, no significant difference was found in Breast cancer frequency between women with minimal or mild background parenchymal enhancement category and normal women (OR, 1.20; 95% CI 0.54–2.67, *p* = 0.66) with high heterogeneity (I^2^ = 96%) as shown in Fig. 4. Stratified analysis of selected studies that did and did not adjust for age, and ethnicity of the women and was not done since no studies reported or adjusted for these elements or whether higher background parenchymal enhancement is associated with all cancers or with the special type of cancer.

Based on the visual inspection of the funnel plot as well as on quantitative measurement using the Egger regression test, there was no evidence of publication bias (*p* = 0.88).

## Discussion

In our meta-analysis study based on 13 studies including 13,788 women at the start with 4046 breast cancer. Women with moderate and high background parenchymal enhancement has up to 2.66-fold higher risk of breast cancer compared to normal women. This effect was detected in all populations [5, 8, 9, 17–26].

Background parenchymal enhancement is chiefly affected by the estrogen hormone. Estrogen hormone affects many systems and organs, including the reproductive system, urinary system, cardiovascular system, bones, skin, hair, and brain. It is believed that background parenchymal enhancement is the radiologically visible form and image of the circulating estrogen hormone in the breast. Besides estrogen hormone, many other factors affect this dynamic enhancement process, such as the amount of contrast material, the patient's hemodynamic status, parameters of magnetic resonance imaging sequences, and vascular anatomy [27].

So, background parenchymal enhancement might be a good tool for improving early discovery and inhibition of breast cancer; since its data is more accessible due to the high number of prescribed breast magnetic resonance imaging for breast cancer screening and diagnosis [12]. A considerable decrease in background parenchymal enhancement was detected post-risk reducing salpingo-oophorectomy [24, 28]. Also, any variations in background parenchymal enhancement were associated with a higher risk of breast cancer, especially in women with breast cancer gene mutation [29]. Therefore, the variations in background parenchymal enhancement measurements may be very effective in forecasting the breast cancer frequency in women with breast cancer gene mutations, especially between women with lower or no hormone levels. Though, our meta-analysis study could not answer if background parenchymal enhancement is associated with higher breast cancer frequency in women with breast cancer gene mutations or not. Furthermore, breasts mostly have a suboptimal examination. The examination is suggested in high-risk women to detect any early potential cancer, which may be responsive to good results [5, 6, 8, 9, 17–25, 29–33].

A cross-sectional study performed on women with normal risk showed a great association between higher levels of background parenchymal enhancement and breast cancer [31]. This recommends that the higher levels of background parenchymal enhancement are more often observed in the contralateral healthy breast of women with suspicious lesions on the other side at magnetic resonance imaging [31].

None of the selected studies reported an association between age, and ethnicity, and breast cancer frequency. Also, none of the studies answered whether higher background parenchymal enhancement is associated with all cancers or with a specific subtype of cancer. However, from the study results presented here, we recommend increasing the use background parenchymal enhancement for detection of the risk of breast cancer to oppose the possible negative outcome as early as possible.

### Limitations

There may be selection bias in this study since so many of the studies found were excluded from the meta-analysis. However, the studies excluded did not satisfy the inclusion criteria of our meta-analysis.

Minimal and mild were represented separately in some studies and together in some studies. So we presented them together here. Maybe if we studied each one alone we could have a significant effect of mild associated with breast cancer but the number of studies that showed them separately was small to conduct a meta-analysis.

## Conclusions

Our meta-analysis showed that the women with high and moderate background parenchymal enhancement at breast magnetic resonance imaging have an association with up to 2.66 fold risk of breast cancer compared to normal women. Background parenchymal enhancement can serve as a tool to improve fast discovery and inhibition of breast cancer. We suggest that women with high or moderate background parenchymal enhancement at breast magnetic resonance imaging to be scheduled for more frequent follow up and screening for breast cancer to avoid any complications.

## Data Availability

The datasets analyzed during the current study are available from the corresponding author on reasonable request.

## Abbreviations

OR: Odds ratio
CIs: Confidence intervals
